# Supplementary material for: Giant Superlinear Power Dependence of Photocurrent Based on Layered Ta2NiS5 Photodetector
Source: Adv Sci (Weinh). 2023 Apr 28;10(20):2300413. doi: 10.1002/advs.202300413 (PMC10369293; doi:10.1002/advs.202300413)
Supplement: Supplementary file 1 — Supporting Information [file ADVS-10-2300413-s001.pdf]

ADVANCED SCIENCE
Open Access

Supporting Information

for *Adv. Sci.*, DOI 10.1002/advs.202300413

Giant Superlinear Power Dependence of Photocurrent Based on Layered Ta_2NiS_5 Photodetector

*Xianghao Meng, Yuhan Du, Wenbin Wu, Nesta Benno Joseph, Xing Deng, Jinjin Wang, Jianwen Ma, Zeping Shi, Binglin Liu, Yuanji Ma, Fangyu Yue, Ni Zhong, Ping-Hua Xiang, Cheng Zhang, Chun-Gang Duan, Awadhesh Narayan, Zhenrong Sun, Junhao Chu and Xiang Yuan**

Supplementary information for

Giant superlinear power dependence of photocurrent based on layered Ta_2NiS_5 photodetector

Xianghao Meng[1,2,†], Yuhan Du[1,2,†], Wenbin Wu[1,2,†], Nesta Benno Joseph[3], Xing Deng[4], Jinjin Wang[4], Jianwen Ma[5], Zeping Shi[1,2], Binglin Liu[1,2], Yuanji Ma[1,2], Fangyu Yue[1,2,4], Ni Zhong[1,2,4], Ping-Hua Xiang[1,2,4], Cheng Zhang[5,6], Chun-Gang Duan[1,2,4], Awadhesh Narayan[3], Zhenrong Sun[1,2], Junhao Chu[2,4,7], Xiang Yuan[1,2*]

[1]State Key Laboratory of Precision Spectroscopy, East China Normal University, Shanghai 200241, China

[2]School of Physics and Electronic Science, East China Normal University, Shanghai 200241, China

[3]Solid State and Structural Chemistry Unit, Indian Institute of Science, Bangalore 560012, India

[4]Key Laboratory of Polar Materials and Devices, Ministry of Education, East China Normal University, Shanghai 200241, China

[5]State Key Laboratory of Surface Physics and Institute for Nanoelectronic Devices and Quantum Computing, Fudan University, Shanghai 200433, China

[6]Zhangjiang Fudan International Innovation Center, Fudan University, Shanghai 201210, China

[7]Institute of Optoelectronics, Fudan University, Shanghai 200438, China

[*]Correspondence and requests for materials should be addressed to X. Y. (E-mail: xyuan@lps.ecnu.edu.cn)

[†]These authors contributed equally to this work.

Table of Content

I. **The spatial profile of the laser spot**
II. **Angle-resolved polarized Raman spectroscopy**
III. **The same fitted values of γ in the log-log plot**
IV. **Exclusion of Bolometric effect**
V. **Two-recombination-center model**
VI. **Infrared Spectroscopy**
VII. **DFT Calculation**
VIII. **Transport Experiments**
IX. **Power-dependent response speed**
X **Superlinearity and beam-size dependency**

I. The spatial profile of the laser spot

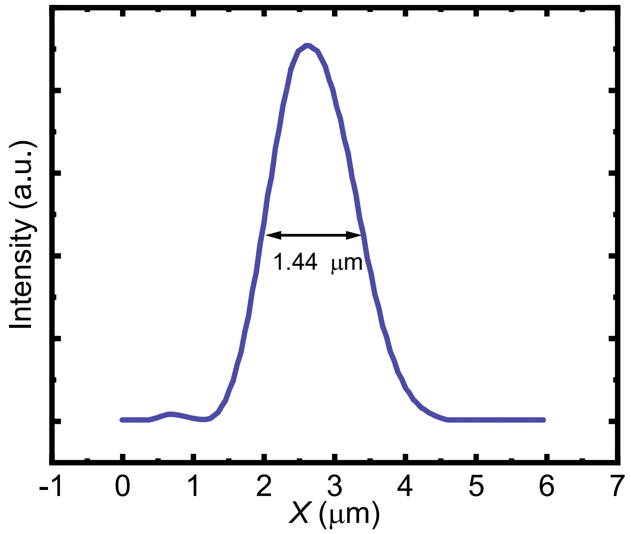

Fig. S1 The irradiance distribution of the laser spot. The full width at half-maximum of the spot is 1.44 μm.

In our experiment, the full width at half-maximum of the laser intensity distribution is 1.44 μm as shown in Fig. S1. It is much smaller compared to the size of the sample and electrodes which ensures sufficient resolution to distinguish the spatial origin of the light response. Based on the photocurrent scanning measurement in main text Fig. 2e, the photocurrent is prominent on the Ta_2NiS_5 channel while negligible elsewhere. Combined with the indispensable working bias, it suggests the photoconductive origin of the Ta_2NiS_5 devices.

II. Angle-resolved polarized Raman spectroscopy

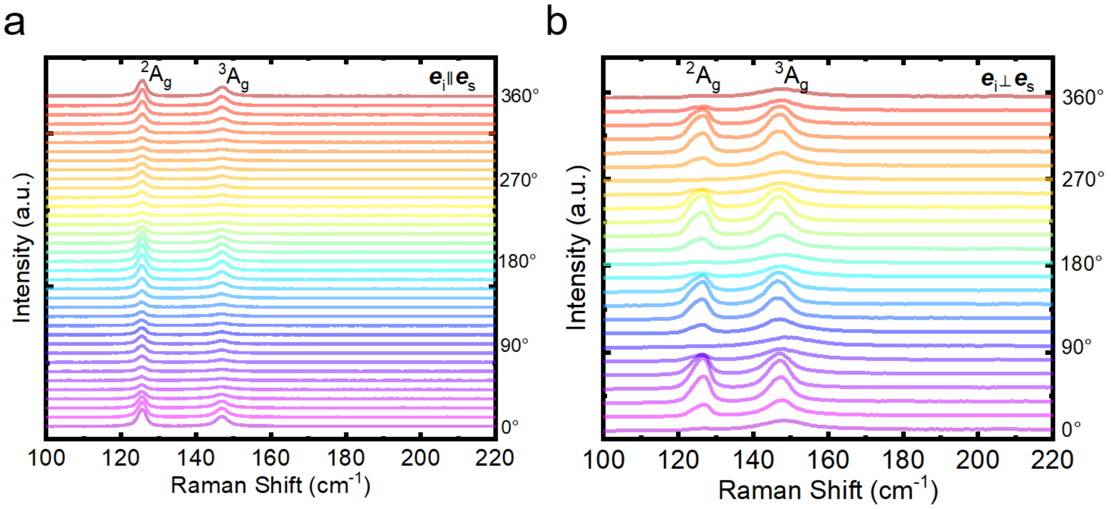

Fig. S2 Angle-resolved polarized Raman spectroscopy. a, b, Raman spectra obtained in parallel and perpendicular configurations.

In order to check the crystal orientation and compare it with the angle-resolved photocurrent measurement, the angle-resolved polarized Raman spectroscopy is carried out as shown in Fig. S3. The labeled angle is defined by the angle between the polarization direction of the incident light and the a-axis (armchair direction) of the sample. The experiment is performed at room temperature under the illumination of HeNe Laser. The intensity of the Raman mode varies periodically with the angle which agrees well with the Raman tensor analysis in the main text.

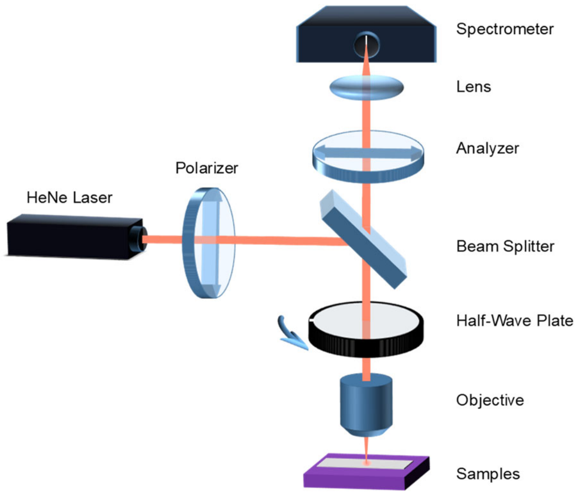

Fig. S3 Experimental setup of the angle-resolved polarized Raman spectrum. The incident angle of the laser is controlled by the half-wave plate. Parallel (perpendicular) polarization configurations are measured with a polarizer parallel (perpendicular) to the analyzer.

III. The same fitted values of γ in the log-log plot

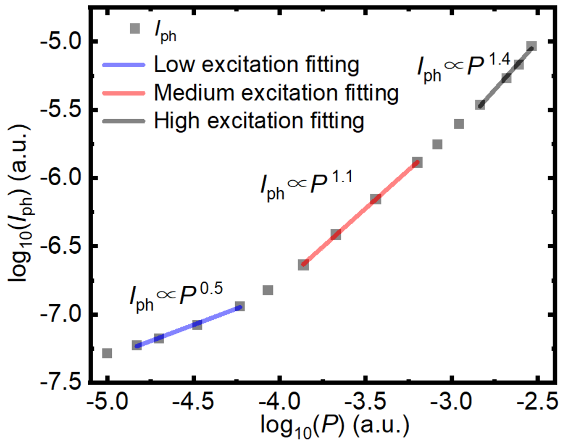

Fig. S4 Log-log plot of photocurrent versus illumination power. The slopes of linear fitting correspond to the power exponent γ in the three different power regimes, respectively. Compared with the values in Fig. 3c-e, they are almost equal in the same regime.

IV. Exclusion of Bolometric effect

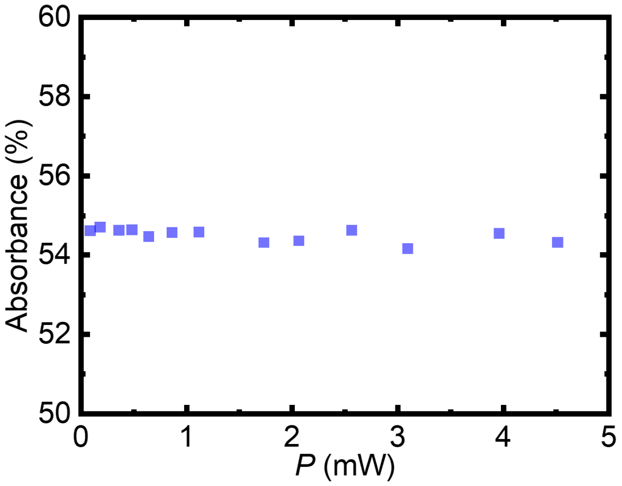

Fig. S5 Absorbance of multi-layer Ta_2NiS_5 under 632.8 nm laser.

The bolometric effect consists of a series of physical processes. Here we analyze the power-dependence for each of the processes. The first one is absorbing the incident light as shown in Fig. S5. We found that absorbance of the multi-layer Ta_2NiS_5 at 632.8 nm keeps invariant at different power and even presents a slightly decreased value at high power. The second process is the temperature rising due to photon absorption. This process is known to be strongly sublinear due to the more efficient cooling at the higher temperature. The third process is the conductivity variation originating from the temperature rising. As shown in Fig. S6, the conductivity increases almost linearly with the temperature near the experimental temperature. This is quantitatively supported by the power law fitting with a fitted power exponent of 1.02. None of the discussed physical processes involving in bolometric effect present superlinear power-dependence in the Ta_2NiS_5 device. Therefore, the observed photoresponse is not expected to come from bolometric effect. Combined with other performance of the device mentioned in the main text, the photoconductive origin is fully supported.

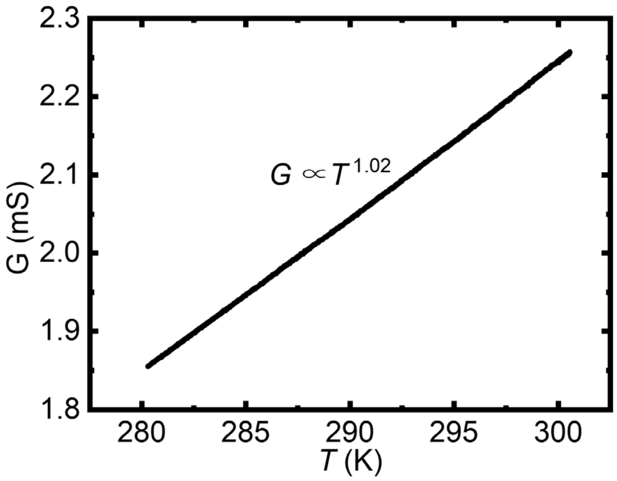

Fig. S6 Electrical conductivity versus temperature of Ta_2NiS_5. The conductivity increases almost linearly with temperature as indicated by the power law fitting.

V. Two-recombination-center model

Table S1 The model parameter of two-RC model

$\boldsymbol{E_1}$	Energy difference between RC_1 and VB	$\boldsymbol{E_2}$	Energy difference between RC_2 and CB
$\boldsymbol{N_i}$	Density of the ith state	$\boldsymbol{n_i}$	Density of the filled ith state
$\boldsymbol{n}$	Density of the photo-generated free electrons	$\boldsymbol{p}$	Density of the photo-generated free holes
$\boldsymbol{S_{n_i}}$	Capture cross-section of the ith state for electrons	$\boldsymbol{S_{p_i}}$	Capture cross-section of the ith state for holes
$\boldsymbol{P_1}$	Probability per unit time for thermal ejection of a hole in RC_1 into the VB	$\boldsymbol{P_2}$	Probability per unit time for thermal ejection of an electron in RC_2 into the CB
$\boldsymbol{F}$	Density of electron-hole pairs created per second by optical excitation across the band gap	$\boldsymbol{v}$	Thermal velocity of the electron and hole (assuming equally for simplicity)
$\boldsymbol{\beta_{n_i}}$	Product of S_{n_i} and v for an electron	$\boldsymbol{\beta_{p_i}}$	Product of S_{p_i} and v for a hole
$\boldsymbol{S'}$	Recombination cross-section between free electrons and free holes	$\boldsymbol{\beta'}$	Product of S' and v for a hole

In a steady state, the overall processes in the main text Fig. 4 can be described by the following equations

$$\frac{dn}{dt} = F - \beta' np - n[\beta_{n_1}(N_1 - n_1) + \beta_{n_2}(N_2 - n_2)] + n_2 P_2 \quad (1)$$

$$\frac{dp}{dt} = F - \beta' np - p[\beta_{p_1} n_1 + \beta_{p_2} n_2] + (N_1 - n_1) P_1 \quad (2)$$

$$\frac{dn_1}{dt} = (N_1 - n_1) P_1 - p\beta_{p_1} n_1 + n\beta_{n_1}(N_1 - n_1) \quad (3)$$

$$\frac{dn_2}{dt} = n\beta_{n_2}(N_2 - n_2) - p\beta_{p_2} n_2 - n_2 P_2 \quad (4)$$

The equation (1) depicts the changing rate of the electron density in conduction band. The F, $-n\beta_{n_1}(N_1 - n_1)$, $-n\beta_{n_2}(N_2 - n_2)$, n_2P_2, and $-\beta' np$ term corresponds to the procedure A, B, C, D, and H, respectively. On the other hand, equation (2) characterizes the changing rate of the hole density in valence band. The $-p\beta_{p_2}n_2$, $-p\beta_{p_1}n_1$, and $(N_1 - n_1)P_2$ term corresponds to the processes of E, G, and F. And the equations (3) and (4) denote the changing rate of the electron density of RC_1 and RC_2.

The photoelectric transition originating from the RC_1 to the conduction band should be allowed upon light illumination but ignored in the model. This is because the DOS of RC is much lower than the conduction and valence band. Therefore, such a process bears a negligibly low probability compared to the cross-gap interband photoelectric transition. Therefore, this process is not considered in the RC model. It is important to note that this assumption is also taken in previous superlinearity photocurrent studies with RC model[1–8].

The RC_1 is below the Fermi level under darkness, so the RC_1 is fully occupied at this condition. Upon illuminating the device, the quasi Fermi level splits which leads to the partial occupation of RC_1[7,8]. Meanwhile, a series of electronic transitions reaches dynamic equilibrium including thermal excitation, trapping, and nonradiative recombination. By increasing the illuminating power, the system shifts to a new equilibrium where the electron occupation of RC_1 is further lowered. The occupation condition agrees with the model and experimental findings.

The superlinear behavior under high-intensity illumination is well explained by the two-RC model. For more quantitative analysis, we fit the power-dependence of experimental photocurrent with the proposed model. Due to the non-linearity of the equation and large number of the fitting parameters, we build a program and perform the fitting by the gradient descent. The convergence of the fitting can be accessed within 24 hours with proper setting of the initial value. The N_1 and N_2 are fitted to be 1.42×10^{18} cm^{-3} and 8.84×10^{16} cm^{-3}, respectively. While S_{n1} and S_{n2} are fitted to be 7.16×10^{-30} cm^2 and 8.26×10^{-26} cm^2, respectively.

The model fits well with the experimental finding. The N_1/N_2 reaches 16, which is large enough for suppressing the C process and leads to the saturation of RC_2. Meanwhile, the S_{n_1}/S_{n_2} is found $< 1 \times 10^{-4}$, small enough to restrain the B process.

VI. Infrared Spectroscopy

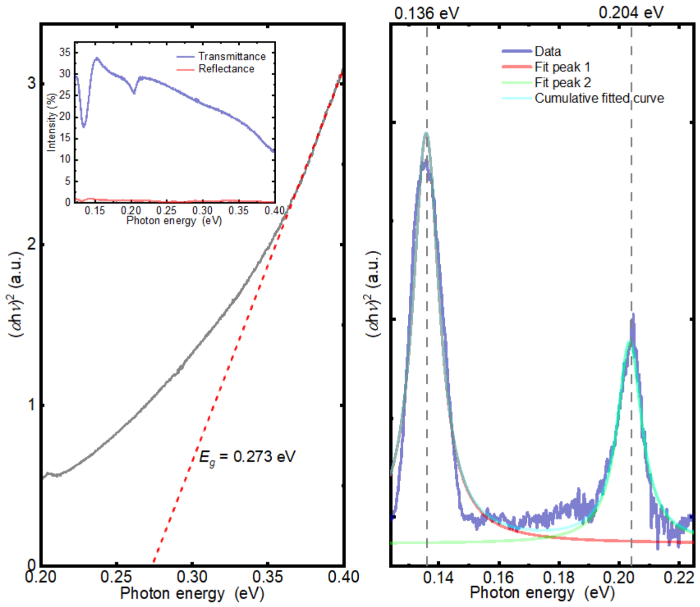

Fig. S7 Infrared spectrum. a, Infrared spectrum and the fitting of the optical band gap $E_g = 0.273$ eV. The inset is the transmission spectrum and reflectance spectrum of Ta_2NiS_5. **b**, Lorentz fitting of the spectral peaks after extracting the spectral background, indicating the presence of the in-gap states.

The transmission spectrum and reflectance spectrum of Ta_2NiS_5 nano-flake are measured by Fourier transform infrared microscope equipped with Mercury Cadmium Telluride detector (Inset of Fig. S7a). The optical transition across the band gap leads to the frequency-dependent absorption following

$$(\alpha h\nu)^m \propto (h\nu - E_g)$$

where h, ν, E_g denotes Planck constant, photon frequency, and band gap, respectively. $m = 2$ and $m = 1/2$ for direct and indirect band gap, respectively. Our infrared spectrum can be fitted by the direct band gap case with $E_g = 273$ meV as presented in Fig. S7a. The extracted gap type and gap size agree with the consensus of Ta_2NiS_5 being a direct and narrow gap semiconductor[9–12].

In addition to the band edge absorption, there are two additional absorbance peaks located at much lower energy than the band gap, indicating the presence of in-gap states and corresponding in-gap optical transitions. The phonon origin of those features can be excluded due to the much higher energy compared to the existing phonon modes [13]. By performing multi-peak Lorentz fitting, the absorbance peaks are found to be located at 136 meV and 204 meV as exhibited in Fig. S7b.

VII. DFT Calculation

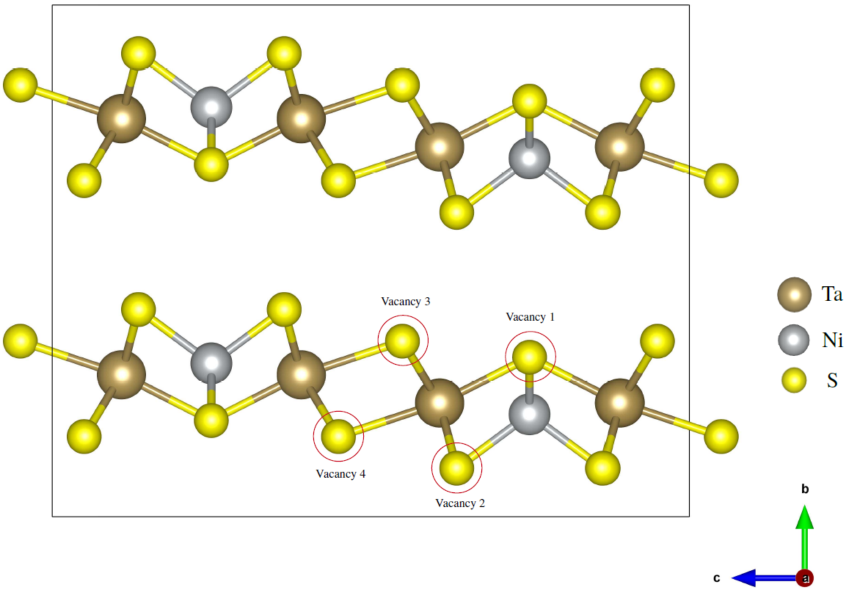

Fig. S8 3 × 1 × 1 supercell of Ta_2NiS_5. The four different S vacancies considered are marked as vacancy 1-4.

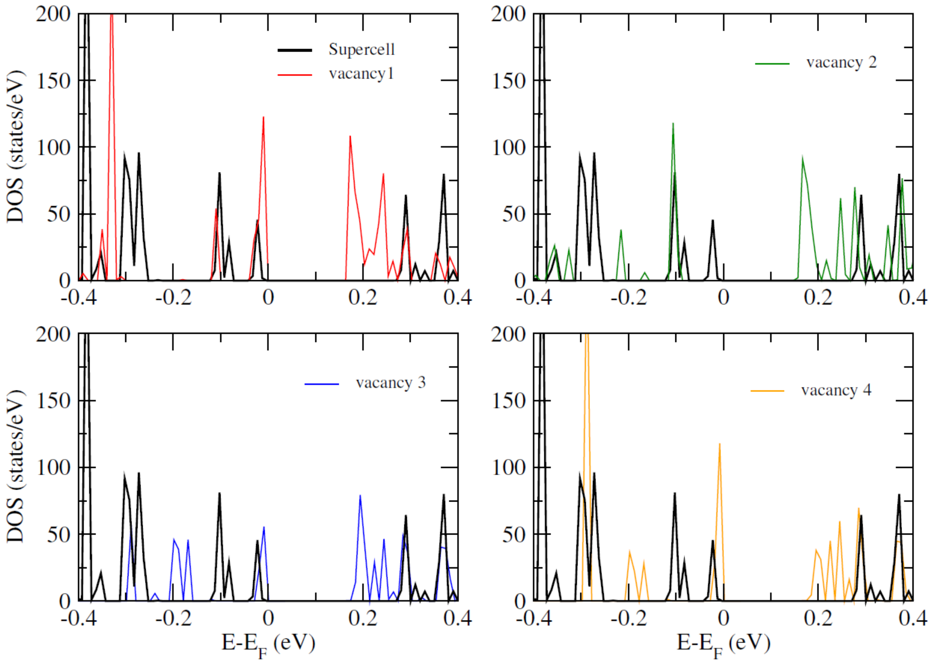

Fig. S9 Density of states associated with different vacancy configurations Ta_2NiS_5 supercell. The black lines belong to pristine supercell, while red, green, blue, and orange lines are associated with the DOS of the four vacancies considered in Fig. S9.

Our first-principle calculations were carried out based on the DFT framework as implemented in the QUANTUM ESPRESSO CODE[14,15]. A kinetic energy cut-off of 60 Ry was considered, using ultrasoft pseudopotentials[16] to describe the core electrons. We used the Perdew-Burke-Ernzerhof (PBE) form of the generalized gradient approximation (GGA) for the exchange-correlation functional[17]. As GGA-

PBE results in a metallic band structure for orthorhombic Ta_2NiS_5, a tiny monoclinic distortion (β = 90.5°) was employed to open up a band gap. Since GGA-PBE is well-known to underestimate the bandgap, we subsequently used a scissor correction[18] to match the experimentally observed band gap of 273 meV. The S vacancies were modeled by using a supercell of $3 \times 1 \times 1$ with four different types of vacancies considered (as shown in Fig. S8). The Brillouin zone was sampled over a uniform Γ-centered *k*-mesh of $2 \times 6 \times 3$.

The resulting DOS associated with these kinds of vacancies are shown in Fig. S9. The black curves are the DOS for the pristine supercell without any vacancies. Each panel shows the comparison of the pristine supercell with the vacancy system. We note that for nearly all vacancy configurations, in-gap states are in the range of $170 - 240$ meV above the valence band maximum. These may be the origin of vacancy states observed in the experimental results. Our calculations also make it plausible that such vacancy-induced in-gap states might be quite abundant in Ta_2NiS_5 system. The experimentally observed lower in-gap states remain not resolved in the current stage of the calculation. Due to the absence of lower in-gap states, the Fermi level is found at the top of the valence band. The lower recombination centers might also originate from other defects such as impurities or dangling bonds [4,19–21]. More calculations for all possible defects need to be carried out in the future for identifying the origin of the in-gap states.

VIII. Transport Experiments

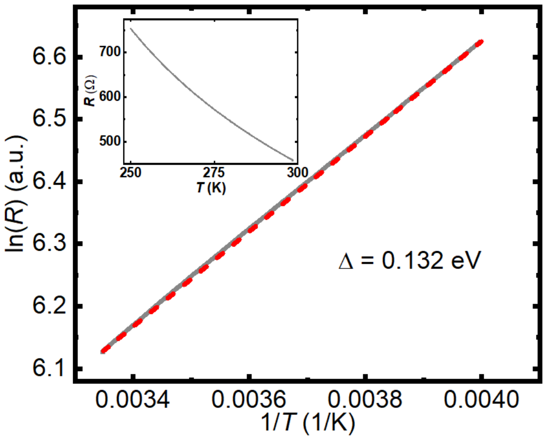

Fig. S10 Transport excitation energy. Excitation energy Δ of Ta_2NiS_5. From the electrical transport experiment, the Δ is extracted as 132 meV, suggesting the influence of the ionized dopants. The red dashed line is the activation fitting curve. Inset is the R-T curve near room temperature.

The gap size and the location of Fermi energy can also be determined by the temperature (T) dependence of the electrical resistance (R). In the inset of Fig. S10, the resistivity was measured by a conventional four-probe setup which drops with increasing temperature. It indicates the activation behavior of semiconductors where the conductivity variation is dominated by conducting carrier density. The activation energy can be extracted by fitting the following equation

$$\sigma \propto \exp\left(-\frac{\Delta}{k_{\mathrm{B}}T}\right).$$

In Fig. S10, the measured conductivity ($\sigma \propto 1/R$) clearly follows the equation, and the activation energy Δ is extracted as 132 meV, which is much smaller than the optical band gap. In the intrinsic semiconductor, the activation energy is expected to be close to the gap size. Thus, the resistivity of the sample is found prominently affected by the impurities. Such substantial deviation might be explained by the strong ionization of dopants at room temperature. Considering the strong ionization case, the temperature-dependent conductivity is derived as [22]

$$\sigma \propto \exp\left(-\frac{E_c - E_F}{k_B T}\right).$$

The fitted value of activation energy corresponds to the distance of the Fermi level with respect to the band edge. Combined with the gap size extracted by the infrared spectrum, the Fermi level is found close to the gap center but may deviate a few meV away from the exact center position.

By gathering all the information from the infrared and transport experiment, the energy position can be estimated, where RC_1, RC_2, Fermi level locates at 137 meV, 204 meV, and 141 meV above the valence band, respectively. The position of Fermi energy agrees with the ionization condition. In addition, the extracted

Fermi energy also agrees with the observed optical activity of the in-gap transitions following Pauli blocking rules. It is worth mentioning that infrared, transport, and photocurrent measurements are all particle-hole insensitive. In other words, the physical behavior would be identical if exchanging the electrons and holes.

IX. Power-dependent response speed

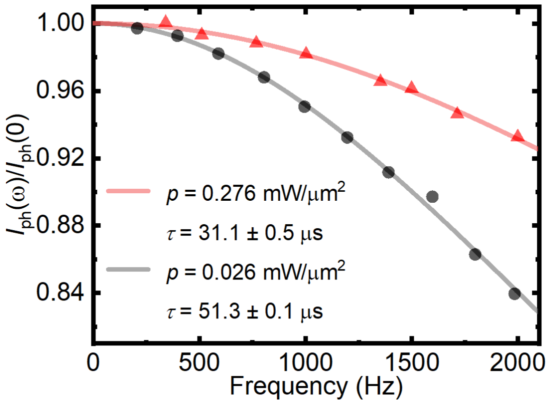

Fig. S11 Frequency-dependent photocurrent measurement at different illuminating power. The response speed is lowered at weaker illumination. Solid lines are the fitting curves.

The recombination process has an impact on the response speed of optoelectronic devices. The comparative fast photoresponse suggests low dopant density, which is in fact, in accordance with our model. Nevertheless, the dopants and resultant in-gap states are indispensable for superlinear photoresponse. However, the strong superlinearity also requires the dopant density as well as the density of states for the in-gap states to be low. Only with a low density of the dopants, the recombination center can be fully occupied at high illuminating power. Otherwise, the existing power of the laser would not be enough to saturate RC_2. The strong superlinearity requires the dopants to be optimized in the low-density regime. Therefore, the comparatively fast photoresponse is reasonable.

A minor contributing factor is that recombination centers are saturated under light illumination and the recombination process is suppressed. Thus, the photoresponse is less influenced by those defects. If so, we might expect the photoresponse speed to become slower at weaker illumination conditions. We perform the frequency-dependent photocurrent measurement with lower incident light. As exhibited in Fig. S11, the photoresponse drops much faster with frequency at lower illuminating power, indicating lower speed. The response time increases from $31.1\ \mu s$ to $51.3\ \mu s$ by reducing the illuminating power density from $0.276\ mW/\mu m^2$ to $0.026\ mW/\mu m^2$ as expected based on the model.

X. Superlinearity and beam-size dependency

The beam-size-dependent experiments might provide another contributing evidence for the superlinearity of the photoresponse. By expanding the beam spot size and maintaining the power of the entire spot, the effective power density is expected to be lowered, resulting in lower photocurrent density (defined as photocurrent per functional area). For linear response devices under the ideal condition, the overall photocurrent is expected to stay constant because the lowered power density will be exactly compensated by the larger photoresponse area. However, for superlinear devices, the photocurrent density variation is also superlinear. Thus, the linearly increased photoresponse area cannot fully compensate for the loss of superlinear photocurrent density, contributing to the drop of overall photocurrent with larger beam spot size. We performed a beam size-dependent experiment on the variation of the photocurrent under constant power but different sizes of the light spot. The overall photocurrent decreases to 2.78 μA and 2.58 μA while the full width at half-maximum of the light spot increases to 3.5 μm and 4.16 μm, respectively.

References

[1] Bube, R. H. Analysis of photoconductivity applied to cadmium-sulfide-type photoconductors. *Journal of Physics and Chemistry of Solids* **1**, 234–248 (1957).

[2] Anomalous Photoconductive Transport Properties of As_2Se_3 Films. *Egyptian Journal of Solids* **25**, 13–21 (2002).

[3] Peters, J. A., Cho, N. K., Liu, Z., Wessels, B. W., Li, H., Androulakis, J. & Kanatzidis, M. G. Investigation of defect levels in $Cs_2Hg_6S_7$ single crystals by photoconductivity and photoluminescence spectroscopies. *Journal of Applied Physics* **112**, 063702 (2012).

[4] Kushwaha, N., Kushwaha, V. S., Shukla, R. K. & Kumar, A. Determination of energy of defect centres in a-$Se_{78}Ge_{22}$ thin films. *Philosophical Magazine Letters* **86**, 691–697 (2006).

[5] Fan, C., Yue, Q., Yang, J., Wei, Z., Yang, S. & Li, J. Low temperature electrical and photo-responsive properties of $MoSe_2$. *Applied Physics Letters* **104**, 202105 (2014).

[6] Zhang, B. Y., Liu, T., Meng, B., Li, X., Liang, G., Hu, X. & Wang, Q. J. Broadband high photoresponse from pure monolayer graphene photodetector. *Nature Communications* **4**, 1811 (2013).

[7] Li, L., Wang, W., Gan, L., Zhou, N., Zhu, X., Zhang, Q., Li, H., Tian, M. & Zhai, T. Ternary Ta_2NiSe_5 Flakes for a High-Performance Infrared Photodetector. *Advanced Functional Materials* **26**, 8281–8289 (2016).

[8] Klee, V., Preciado, E., Barroso, D., Nguyen, A. E., Lee, C., Erickson, K. J., Triplett, M., Davis, B., Lu, I.-H., Bobek, S., McKinley, J., Martinez, J. P., Mann, J., Talin, A. A., Bartels, L. & Léonard, F. Superlinear Composition-Dependent Photocurrent in CVD-Grown Monolayer $MoS_{2(1-x)}Se_{2x}$ Alloy Devices. *Nano Letters* **15**, 2612–

2619 (2015).

[9] Larkin, T. I., Yaresko, A. N., Pröpper, D., Kikoin, K. A., Lu, Y. F., Takayama, T., Mathis, Y.-L., Rost, A. W., Takagi, H., Keimer, B. & Boris, A. V. Giant exciton Fano resonance in quasi-one-dimensional Ta_2NiSe_5. *Physical Review B* **95**, 195144 (2017).

[10] Li, L., Gong, P., Wang, W., Deng, B., Pi, L., Yu, J., Zhou, X., Shi, X., Li, H. & Zhai, T. Strong In-Plane Anisotropies of Optical and Electrical Response in Layered Dimetal Chalcogenide. *ACS Nano* **11**, 10264–10272 (2017).

[11] Windgätter, L., Rösner, M., Mazza, G., Hübener, H., Georges, A., Millis, A. J., Latini, S. & Rubio, A. Common microscopic origin of the phase transitions in Ta_2NiS_5 and the excitonic insulator candidate Ta_2NiSe_5. *npj Computational Materials* **7**, 210 (2021).

[12] Canadell, E. & Whangbo, M. H. Metallic versus nonmetallic properties of ternary chalcogenides: tantalum metal selenide, Ta_2MSe_7 (M = nickel, platinum), and tantalum nickel chalcogenide, Ta_2NiX_5 (X = sulfide, selenide). *Inorganic Chemistry* **26**, 3974–3976 (1987).

[13] Larkin, T. I., Dawson, R. D., Höppner, M., Takayama, T., Isobe, M., Mathis, Y.-L., Takagi, H., Keimer, B. & Boris, A. V. Infrared phonon spectra of quasi-one-dimensional Ta_2NiSe_5 and Ta_2NiS_5. *Physical Review B* **98**, 125113 (2018).

[14] Giannozzi, P., Andreussi, O., Brumme, T., Bunau, O., Buongiorno Nardelli, M., Calandra, M., Car, R., Cavazzoni, C., Ceresoli, D., Cococcioni, M., Colonna, N., Carnimeo, I., Dal Corso, A., de Gironcoli, S., Delugas, P., DiStasio, R. A., Ferretti, A., Floris, A., Fratesi, G., Fugallo, G., Gebauer, R., Gerstmann, U., Giustino, F., Gorni, T., Jia, J., Kawamura, M., Ko, H.-Y., Kokalj, A., Küçükbenli, E., Lazzeri, M., Marsili, M., Marzari, N., Mauri, F., Nguyen, N. L., Nguyen, H.-V., Otero-de-la-Roza, A., Paulatto, L., Poncé, S., Rocca, D., Sabatini, R., Santra, B., Schlipf, M., Seitsonen, A. P., Smogunov, A., Timrov, I., Thonhauser, T., Umari, P., Vast, N., Wu, X. & Baroni, S. Advanced capabilities for materials modelling with Quantum ESPRESSO. *Journal of Physics: Condensed Matter* **29**, 465901 (2017).

[15] Giannozzi, P., Baroni, S., Bonini, N., Calandra, M., Car, R., Cavazzoni, C., Ceresoli, D., Chiarotti, G. L., Cococcioni, M., Dabo, I., Dal Corso, A., de Gironcoli, S., Fabris, S., Fratesi, G., Gebauer, R., Gerstmann, U., Gougoussis, C., Kokalj, A., Lazzeri, M., Martin-Samos, L., Marzari, N., Mauri, F., Mazzarello, R., Paolini, S., Pasquarello, A., Paulatto, L., Sbraccia, C., Scandolo, S., Sclauzero, G., Seitsonen, A. P., Smogunov, A., Umari, P. & Wentzcovitch, R. M. QUANTUM ESPRESSO: a modular and open-source software project for quantum simulations of materials. *Journal of Physics: Condensed Matter* **21**, 395502 (2009).

[16] Vanderbilt, D. Soft self-consistent pseudopotentials in a generalized eigenvalue formalism. *Physical Review B* **41**, 7892–7895 (1990).

[17] Perdew, J. P., Burke, K. & Ernzerhof, M. Generalized Gradient Approximation Made Simple. *Physical Review Letters* **77**, 3865–3868 (1996).

[18] Fiorentini, V. & Baldereschi, A. Dielectric scaling of the self-energy scissor

operator in semiconductors and insulators. *Physical Review B* **51**, 17196–17198 (1995).

[19] Jasenek, A. & Rau, U. Defect generation in Cu(In,Ga)Se_2heterojunction solar cells by high-energy electron and proton irradiation. *Journal of Applied Physics* **90**, 650–658 (2001).

[20] Massé, G. & Redjai, E. Radiative recombination and shallow centers in $CuInSe_2$. *Journal of Applied Physics* **56**, 1154–1159 (1984).

[21] Li, J., Yuan, Z.-K., Chen, S., Gong, X.-G. & Wei, S.-H. Effective and Noneffective Recombination Center Defects in Cu_2ZnSnS_4: Significant Difference in Carrier Capture Cross Sections. *Chemistry of Materials* **31**, 826–833 (2019).

[22] Zekry, A. *electronic devices*. (LAP LAMBERT Academic Publishing, 1998, p42-43).
